# Perspectives on implementing HIIT interventions for service users in inpatient mental health settings: a qualitative study investigating patient, carer and staff attitudes

**DOI:** 10.1192/bjo.2021.716

**Published:** 2021-06-18

**Authors:** Rebecca Martland, Juliana Onwumere, Fiona Gaughran, Brendon Stubbs

**Affiliations:** 1 Institute of Psychiatry, Psychology and Neuroscience (IoPPN), Department of Psychosis Studies; 2 Institute of Psychiatry, Psychology and Neuroscience (IoPPN), Department of Psychology,National Psychosis Unit, South London and Maudsley NHS Foundation Trust; 3 Institute of Psychiatry, Psychology and Neuroscience (IoPPN), Department of Psychosis Studies, National Psychosis Unit, South London and Maudsley NHS Foundation Trust; 4 Institute of Psychiatry, Psychology and Neuroscience (IoPPN), Department of Psychological Medicine, South London and Maudsley NHS Foundation Trust

## Abstract

**Aims:**

High intensity interval training (HIIT) may improve a range of physical and mental health outcomes among people with severe mental illnesses (SMI). However, there is limited data on patients’ reported attitudes towards HIIT and its implementation within inpatient settings, and there remains an absence of data on attitudes towards HIIT from informal family carers of service users and healthcare professionals, who both have key roles to play in facilitating recovery outcomes in service users. This study sought to qualitatively investigate, in inpatients with SMI, carer and staff groups, perspectives on implementing HIIT interventions for patient groups in inpatient settings.

**Method:**

Seven focus groups and one individual interview were conducted. These included three focus groups held with inpatients with SMI (n = 12), two held with informal carers (n = 15), and two held with healthcare professionals working in inpatient settings (n = 11). An additional individual interview was conducted with one patient participant. The focus group schedule comprised open- ended questions designed to generate discussion and elicit opinions surrounding the introduction of HIIT on inpatient mental health wards. Data were subject to a thematic analysis.

**Result:**

Two key themes emerged from the data, across all participants, that reflected the ‘Positivity’ in the application of HIIT interventions in psychiatric inpatient settings with beliefs that it would help patients feel more relaxed, build their fitness, and provide a break from the monotony of ward environment. Moreover, the short length of HIIT sessions was deemed appealing to mitigate against difficulties that many inpatients can experience with motivation, interest and attention, and was considered to be more appealing than more lengthy forms of exercise, which may require greater physical exertion. The second theme related to ‘Implementation concerns’, that reflected subthemes about i) low patient motivation, particularly with older participants, those administered many medications, and for those with less positive memories of exercise ii) patient safety, including concerns surrounding the intensity of HIIT and inclusion of patients with physical health comorbidities and iii) practical logistical factors, including having access to the right sports clothing and staff availability to supervise HIIT.

**Conclusion:**

HIIT for inpatients with SMI was actively endorsed by patients, carers and healthcare professionals. Patient safety and baseline motivation levels, and practical service considerations were all noted as potential barriers to successful implementation and are worth considering in preparation for trialing a new intervention.

